# Sinobronchial allergic mycosis syndrome in an elderly male

**DOI:** 10.1186/s13223-019-0349-y

**Published:** 2019-06-04

**Authors:** Eisuke Mochizuki, Shun Matsuura, Tsutomu Kubota, Yasutaka Mochizuka, Kyohei Oishi, Hyogo Naoi, Masahiro Uehara, Shinichiro Mikura, Miyuki Nagaoka, Masaru Tsukui, Naoki Koshimizu, Ichirota Nameki

**Affiliations:** 1 Department of Respiratory Medicine, Fujieda Municipal Hospital, 4-1-11 Surugadai, Fujieda, 426-8677 Japan; 2 Department of Otological Medicine, Fujieda Municipal Hospital, 4-1-11 Surugadai, Fujieda, 426-8677 Japan

## Abstract

**Background:**

Allergic bronchopulmonary aspergillosis (ABPA) and allergic fungal rhinosinusitis (AFRS) are characterized by hyper-responsiveness of the respiratory tract and the nasal cavity and paranasal sinuses, respectively to *Aspergillus* species and AFRS causes chronic rhinosinusitis. Herein, we report the first case of sinobronchial allergic mycosis (SAM) syndrome, defined as ABPA with concomitant AFRS, caused by *Aspergillus fumigatus* patient > 80 years.

**Case presentation:**

An 82-year-old male with interstitial pneumonia who returned for follow-up exhibited high-attenuation mucus plug in the right intermediate bronchial trunk, infiltration in the right lung field, and right pleural effusion on regular chest computed tomography (CT). We found unilateral central bronchiectasis in the right upper lobe. Similarly, CT scan of the paranasal sinuses revealed high-attenuation mucus plugs in left ethmoid sinuses. Biopsy specimens from the plugs in the right intermediate bronchial trunk and the left ethmoid sinuses revealed allergic mucin with layers of mucus eosinophils, eosinophil-predominant mixed inflammatory cell infiltrate and *Aspergillus* hyphae. The patient fulfilled all the major criteria for ABPA and AFRS, and was diagnosed with SAM syndrome. CT scan of the lung and paranasal sinuses revealed apparent amelioration after oral steroid therapy.

**Conclusion:**

Despite mostly reported in relatively young patients, SAM syndrome can occur in elderly individuals as well.

## Background

*Aspergillus* species can cause various allergic diseases, such as allergic bronchopulmonary aspergillosis (ABPA) and allergic fungal rhinosinusitis (AFRS). Concurrent ABPA and AFRS are defined as sinobronchial allergic mycosis (SAM) syndrome [1]. Because ABPA and AFRS are treated by pulmonologists and otolaryngologists respectively, the condition outside of their expertise and coexistence of ABPA and AFRS can be overlooked. Patients with SAM syndrome in previous reports were relatively young. In Japan, the median patient age at ABPA onset is 57 years, which is older than that reported in other countries [2], raising the possibility of patients with SAM syndrome among the elderly generation in Japan. We herein report the case of an 82-year-old male who simultaneously developed ABPA and AFRS and was diagnosed with SAM syndrome.

## Case report

**Fig. 1:**
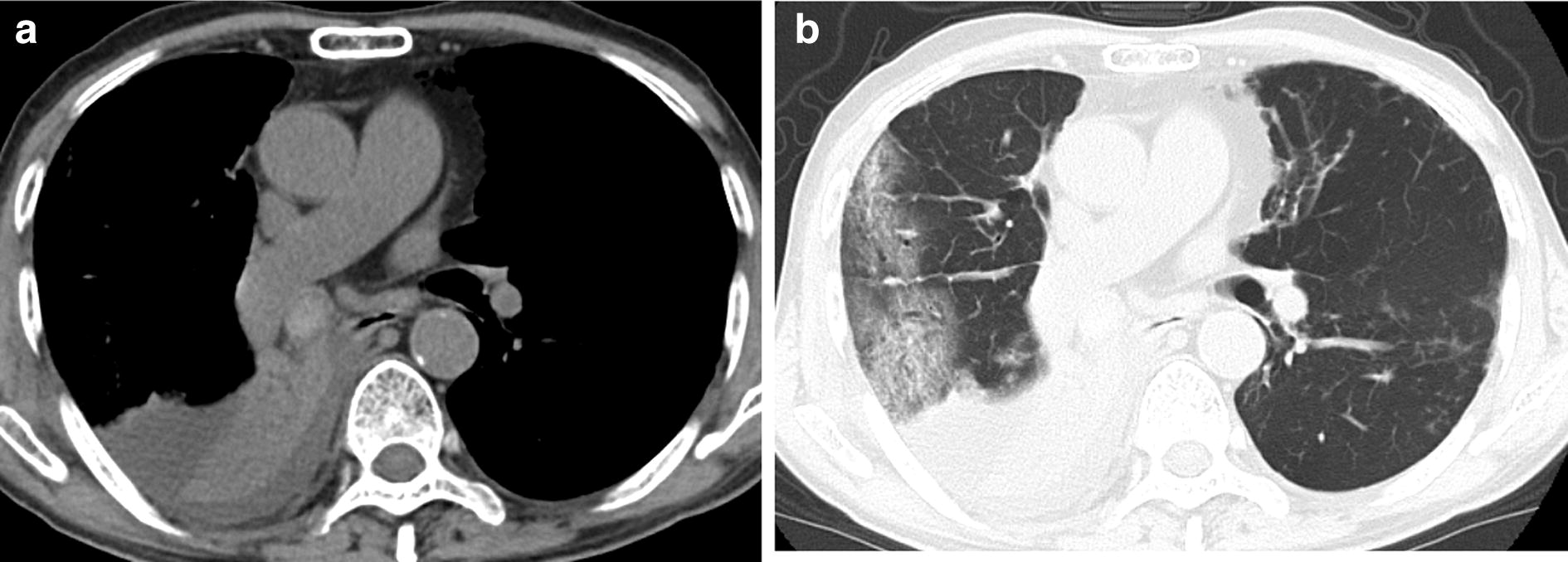
Computed tomography of chest. **a** High attenuation mucus plug in right intermediate bronchial trunk, and right pleural effusion. **b** Infiltration in right lung field

An 82-year-old male was diagnosed with bronchial asthma at 10 years of age and treated with inhalants and he did not receive treatment for asthma since the age of 30 because of the resolution of bronchial asthma. He was followed at our hospital for idiopathic interstitial pneumonia by annual regular chest X-ray and computed tomography (CT) scans for 4 years. The patient’s interstitial pneumonia, with minimal change in radiological findings was stable over the years, he complained no symptoms. He did not indicate exposure to fungus. Chest X-ray and CT scan obtained in December 2017 revealed high-attenuation mucus plug in right intermediate bronchial trunk, right pleural effusion (Fig. 1a), and infiltration in the right lung field (Fig. 1b).

We also found unilateral bronchiectasis in the right upper lobe.

**Fig. 2:**
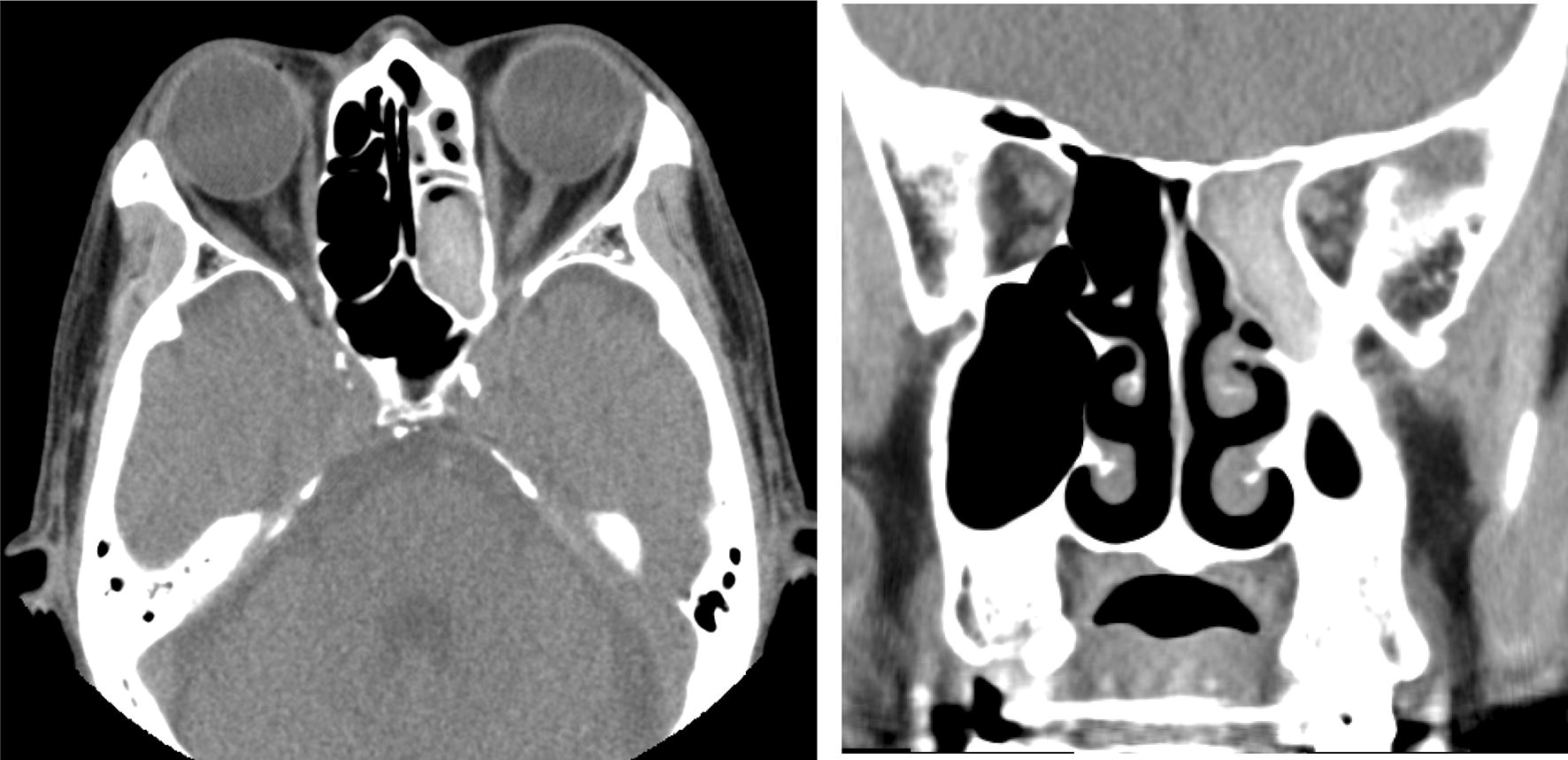
Computed tomography of paranasal sinuses revealing high-attenuation mucus plug in the left ethmoid sinus similar to that observed in the right intermediate bronchial trunk

Additionally, a slight fibrotic change along the pleural line reflecting interstitial pneumonia was observed. CT of paranasal sinuses obtained to investigate nasal congestion for 3 years, revealed high-attenuation mucus plug in the left ethmoid sinuses (Fig. 2). Physical examination revealed decreased breath sounds in the right lower lung field. No wheezing and rhonchi were observed on auscultation.

Blood tests showed a total leucocyte count of 9300/mm^3^ with 8% eosinophils (absolute eosinophil count, 744/mm^3^) and elevated C-reactive protein. Radioimmunosorbent test revealed elevated IgE levels (1460 IU/ml), and the radioimmunosorbent test for specific IgE antibodies against *Aspergillus*, *Penicillium* and *Candida* were positive. Serum precipitins to *Aspergillus* were also positive. We confirmed local urticaria and lash 15 min after subcutaneous injection of *A. fumigatus* antigen and this was positive of immediate cutaneous hypersensitivity reaction. He had a history of right nephrectomy because of renal cancer and did not experience recurrence.

**Fig. 3:**
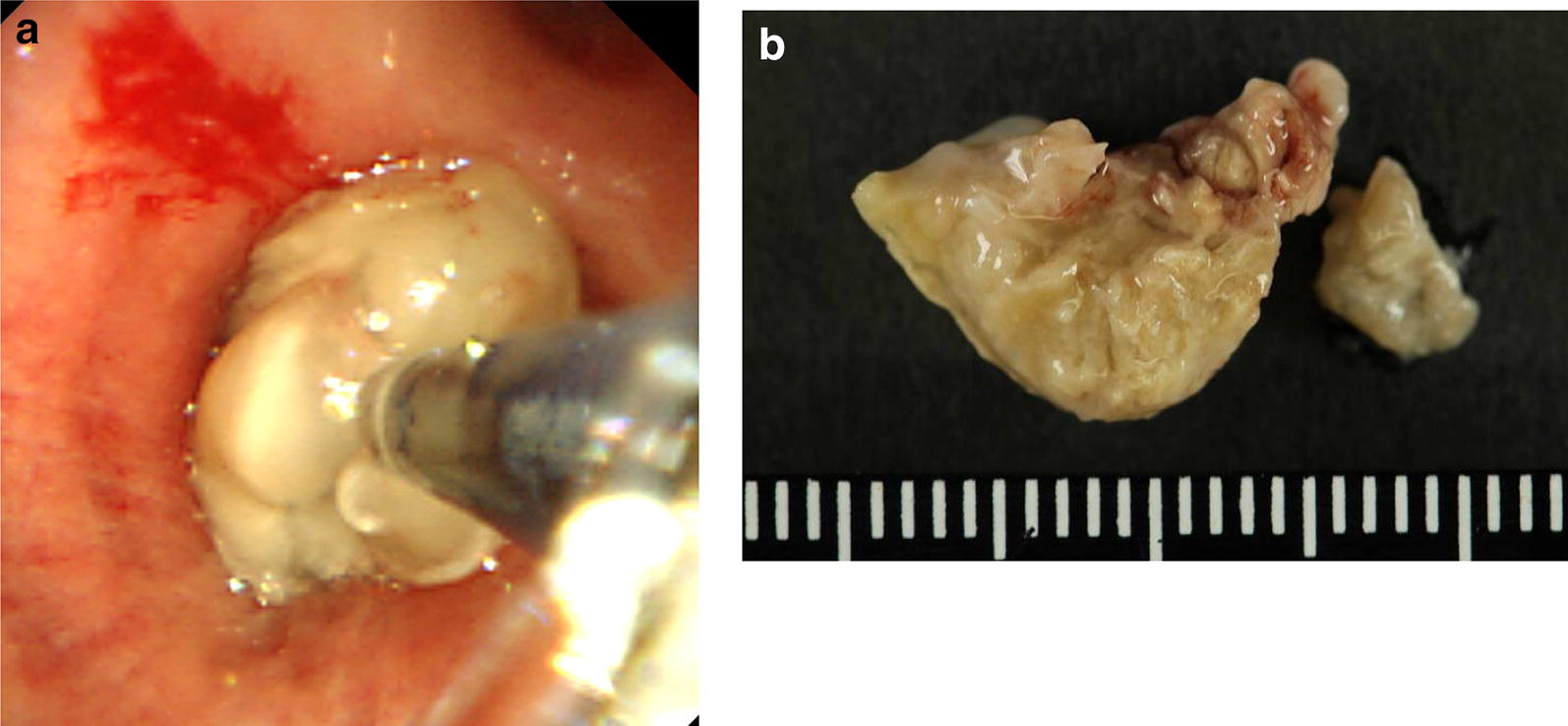
**a** Bronzed, hard, sticky mucus plug in the right intermediate bronchial trunk, which was challenging to retrieve. Multiple biopsy specimens collected from the mucus plug, causing slight bleeding from the bronchial wall. **b** Bronzed, hard, sticky mucus plug in the left ethmoid sinuses

**Fig. 4:**
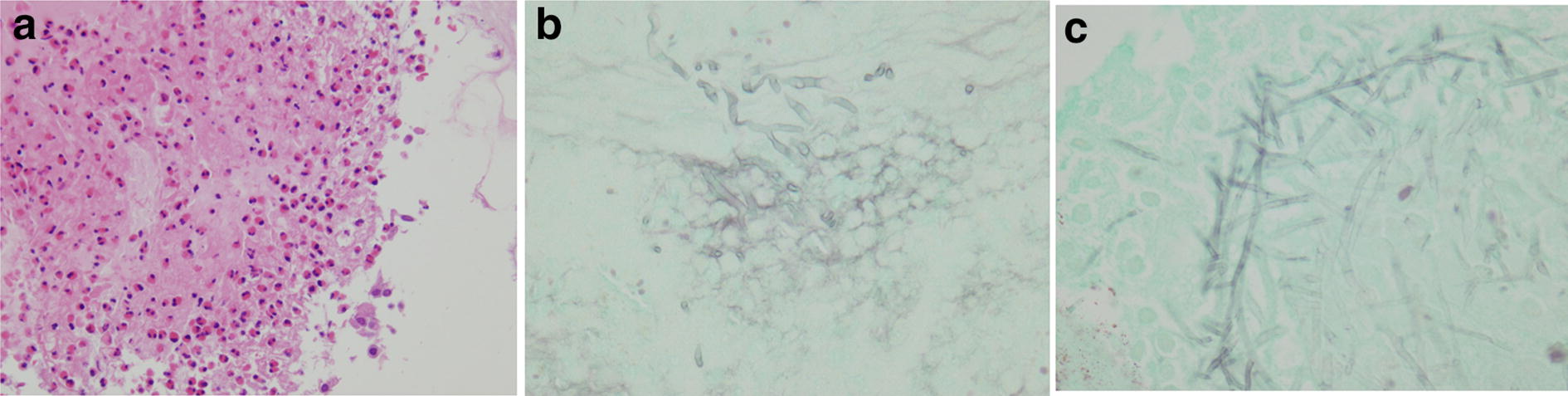
**a** Hematoxylin–eosin staining of allergic mucin with layers of mucus, eosinophils, eosinophil-predominant mixed inflammatory cell infiltrate and Charcot–Leyden crystals. Original magnification, ×200. **b** Grocott’s staining showing fungal hyphae with a 45° branch angle within allergic mucin. Original magnification, ×400. **c** Grocott’s staining showing fungal hyphae within the maxillary sinus. Original magnification, ×400

Pulmonary function test showed the following: forced vital capacity (FVC), 1.86 L (55.4% of predicted value); forced expiratory volume in 1 s (FEV1), 1.60 L (64.3% of predicted value); and FEV1/FVC, 86.0%. Bronchoscopy for definite diagnosis confirmed bronze-colored, hard mucus plug in the right intermediate bronchial trunk (Fig. 3a), and pathological examination of multiple biopsy specimens stained by hematoxylin–eosin revealed allergic mucin with layers of mucus, eosinophils, and eosinophil-predominant mixed inflammatory cell infiltrate and Charcot–Leyden crystals (Fig. 4a). Grocott’s stain revealed fungal hyphae with a 45° branch angle within the allergic mucin (Fig. 4b). We performed open biopsy of the ethmoid sinuses and found similar mucus plug there, which revealed similar pathological findings in the right intermediate bronchial trunk (Fig. 3b), including fungal hyphae with a 45° branch angle within allergic mucin (Fig. 4c). Mucus plug cultures were positive for *A. fumigatus*. The patient met of the diagnostic criteria for ABPA by Rosenberg and Patterson [3] as well as those for AFRS [4], and thus he was diagnosed with SAM syndrome [1].

**Fig. 5:**
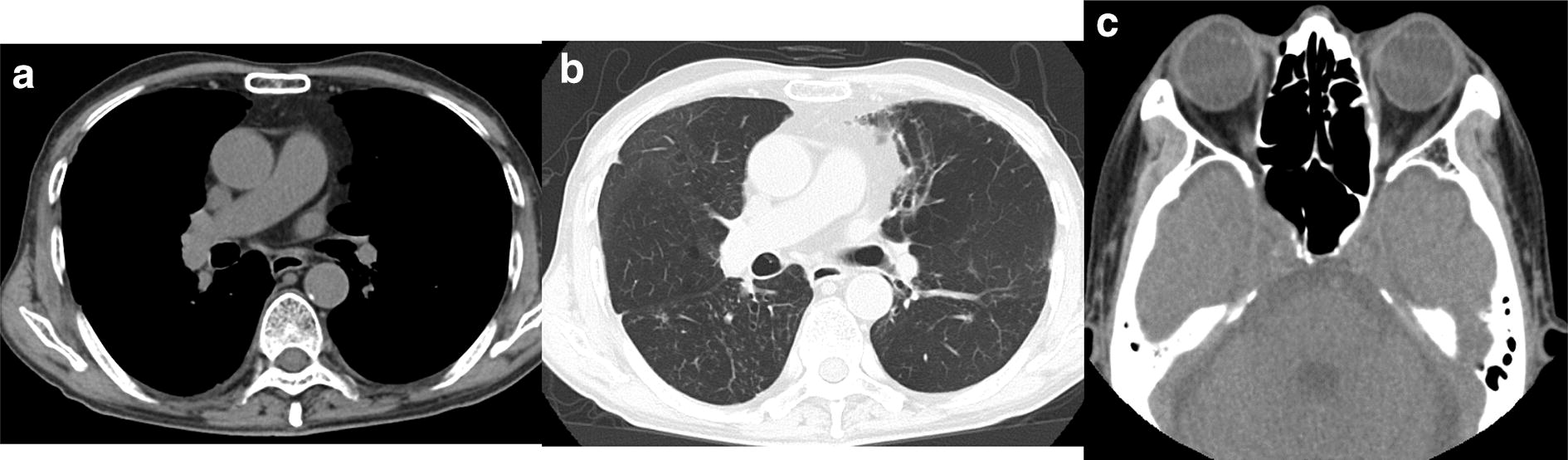
**a**–**c** Computed tomography scan of the lung and paranasal sinuses showing apparent amelioration with treatment

The patient was treated with 0.5 mg/kg prednisolone daily for 1 month, which was reduced to 0.35 mg/kg prednisolone daily. His total IgE levels fell to 206 IU/ml after 3 months. CT of the lung and paranasal sinuses showed apparent amelioration (Fig. 5a–c), and nasal congestion was resolved after 3 months steroid therapy. Now, there has been about 1 year and 4 months after the diagnosis, the patient is currently receiving oral steroid therapy (0.1 mg/kg) and remains in good condition with no deteriorations.

## Discussion

We herein present an elderly patient with a history of bronchial asthma who was diagnosed with SAM syndrome, based on concomitant ABPA and AFRS. Biopsy specimens from the right intermediate bronchial trunk and the left ethmoid sinuses revealed *Aspergillus* hyphae with allergic mucin and extensive eosinophilic infiltration. Oral steroid therapy was efficient against both ABPA and AFRS, and the patient remains in good condition.

ABPA is caused by hyper-responsiveness of the respiratory epithelium to *A. fumigatus*, which was first described in 1952 [5]. In 1977, Rosenberg and Patterson proposed the following diagnostic criteria for ABPA: (1) episodic bronchial obstruction, (2) peripheral eosinophilia, (3) positive immediate skin test to *Aspergillus*, (4) positive precipitin test to *Aspergillus*, (5) increased total serum IgE, (6) history of transient or fixed lung infiltrates, and (7) proximal bronchiectasis [3]. The current patient met all these diagnostic criteria and diagnosed with ABPA. Conversely, AFRS is a distinct type of chronic rhinosinusitis that occurs because of immunological reaction to noninvasive fungi present in the sinuses [6] and can be considered as an upper-airway equivalent of ABPA [1]. In 2004, Meltzer et al. [4] proposed the following diagnostic criteria for AFRS: (1) endoscopic evidence of allergic mucin (pathology showing fungal hyphae with degranulating eosinophils) and sinus inflammation, (2) CT or magnetic resonance imaging findings consistent with rhinosinusitis, (3) evidence of fungal sensitization by skin testing or serum IgE, (4) no histologic evidence of invasive disease. The current patient met the diagnostic criteria of AFRS as well.

Concurrence of ABPA and AFRS is increasingly recognized. In one study of 95 patients with ABPA, diagnoses of definite and probable AFRS were reached in 7 (7%) and 13 (14%) patients, respectively [7], although their concurrence might be more frequent based on the “one airway, one disease” hypothesis. Another study reported that AFRS was confirmed in 80% with patients of ABPA [8]. The difference in the percentage of patients with concomitant ABPA and AFRS between the two studies might be partially explained by the differences in the diagnostic criteria for ABPA and AFRS. SAM syndrome might be more frequent than anticipated. As a rationale for the oversight of their concurrence, Agarwal et al. [9] suggested that different specialists treat ABPA and AFRS, and the use of oral glucocorticoids and/or antifungal agents in either disorder might mask the manifestation of the other disorder.

Treatment approaches for ABPA and AFRS differ to some extent. Therefore, recognition of both disease is important. The cornerstone of ABPA management includes initiation of anti-inflammatory therapy (with systemic glucocorticoids) that aims to attenuate immunological hyperactivity. Systemic glucocorticoids are considered as the treatment of choice. Another treatment option is to use antifungal agents, (such as azoles and nebulized amphotericin B) to attenuate fungal burden in the tracheobronchial tree, thereby decreasing the antigen load [9]. Conversely, the first choice of treatment for AFRS is surgical debridement of the sinuses with endoscopic surgery [10]. Surgery is followed by aggressive medical therapy with postoperative glucocorticoids (prednisolone 0.5 mg/kg for 4 weeks, tapered over the next 2–5 months) to achieve a satisfactory long-term outcome [11]. Agarwal et al. [9] reported that concomitant occurrence of the two disorders is associated with worse outcomes. Therefore, diagnosis should be carefully achieved. In the current case, 0.5 mg/kg oral prednisolone was administered after surgery and the CT scans of the lung and paranasal sinuses demonstrated apparent amelioration.

Of note, the current patient was older than the previously reported patients with SAM syndrome. A Japanese nationwide survey determined that the median age at the onset for ABPA with central bronchiectasis was 57 years, which is older than that reported in other countries [2]. The median ages of onset for ABPA with central bronchiectasis were 34 and 36 years in two Indian studies, 41 years in a Chinese study, and 47 years in a British study [12–15]. We speculate that the onset of SAM syndrome might be later in Japan because of the later onset of ABPA. Moreover, several reports from East Asian countries demonstrate relatively lower total serum IgE levels in patients with ABPA [16–18].

It is conceivable that sensitization to *A. fumigatus* might not decrease with age in asthmatic patients [19]. Tanaka et al. reported that patients with increased IgE over a 10 year period had a higher prevalence of *Aspergillus* sensitization [20], suggesting that the significance of aspergillosis in asthmatic patients might be increasing with age.

Moreover, it is reported that de novo sensitization to *A. fumigatus* in adult asthma over a 10-year observation period [21]. In the study, frequencies of patients positive for IgE to *A. fumigatus* extract increased as the patients get old.

Population aging is a global issue that is estimated to be more rapid in Asian countries. In 2007, more than half of the world’s population was living in cities. Prolonged life expectancy and urbanization are thought to increase asthma in the elderly [22], which is also true for other allergic diseases. Although allergies have been considered a minor concern in the elderly (usually defined as those aged ≥ 65 years), recent epidemiologic studies indicate that allergic diseases are more prevalent than expected in the aged population [23]. Therefore, SAM syndrome should also be considered in aged individuals.

## Conclusion

First case report of SAM syndrome in a patient ≥ 80 years highlights the importance of investigation of paranasal sinusitis for potential AFRS before treatment for ABPA because the treatment approaches for the two diseases are different.

## Data Availability

Please contact author for data requests.

## Abbreviations

ABPA: allergic bronchopulmonary aspergillosis
AFRS: allergic fungal rhinosinusitis
SAM: sinobronchial allergic mycosis
CT: computed tomography
IgE: immunoglobulin E
FEV1: forced expiratory volume in 1 s
FVC: forced vital capacity
